# Extracorporeal membrane oxygenation for acute respiratory distress syndrome

**DOI:** 10.1186/s40560-015-0082-7

**Published:** 2015-06-17

**Authors:** Toshiyuki Aokage, Kenneth Palmér, Shingo Ichiba, Shinhiro Takeda

**Affiliations:** ECMO Centre Karolinska, Astrid Lindgren Children’s Hospital, Karolinska University Hospital, 17176 Stockholm, Sweden; Department of Community and Emergency Medicine, Okayama University Graduate School of Medicine, Dentistry, and Pharmaceutical Sciences, 2-5-1 Shikata-cho, Kita-ku, Okayama 700-8558 Japan; Department of Intensive Care Medicine, Nippon Medical School Hospital, 1-1-5 Sendagi, Bunkyo-ku, Tokyo 113-8603 Japan

**Keywords:** Extracorporeal life support, Extracorporeal membrane oxygenation, Acute respiratory distress syndrome, Hypoxia

## Abstract

Extracorporeal membrane oxygenation (ECMO) can be a lifesaving therapy in patients with refractory severe respiratory failure or cardiac failure. Severe acute respiratory distress syndrome (ARDS) still has a high-mortality rate, but ECMO may be able to improve the outcome. Use of ECMO for respiratory failure has been increasing since 2009. Initiation of ECMO for adult ARDS should be considered when conventional therapy cannot maintain adequate oxygenation. ECMO can stabilize gas exchange and haemodynamic compromise, consequently preventing further hypoxic organ damage. ECMO is not a treatment for the underlying cause of ARDS. Because ARDS has multiple causes, the diagnosis should be investigated and treatment should be commenced during ECMO. Since ECMO is a complicated and high-risk therapy, adequate training in its performance and creation of a referring hospital network are essential. ECMO transport may be an effective method of transferring patients with severe ARDS.

## Introduction

Extracorporeal membrane oxygenation (ECMO) can be employed to salvage patients with refractory severe respiratory failure or cardiac failure. When used for patients with respiratory disease, it is termed respiratory ECMO. Acute respiratory distress syndrome (ARDS) is characterized by acute widespread pulmonary inflammation due to various causes such as viral infection, bacterial infection, trauma, and inhalation of toxic substances. Even in recent years, severe ARDS has a high-mortality rate [1]. However, it has been suggested that ECMO can be employed to improve the outcome. Although the first adult respiratory failure patient treated with ECMO was reported as long ago as 1972, the number of respiratory ECMO patients remained small for more than 30 years afterward [2-4]. While use of ECMO has been increasing in recent years, its indications and clinical management protocols are still under investigation.

## Review

### Principles of ECMO and pathophysiology of ARDS

#### Principles of respiratory ECMO

**Figure 1:**
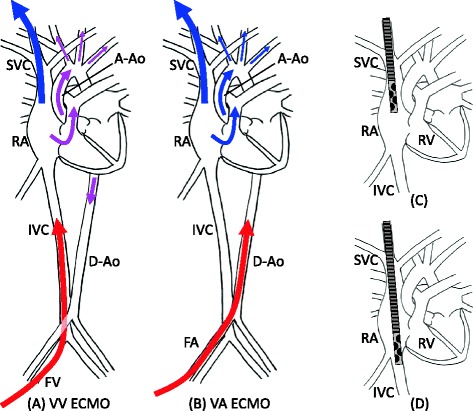
**Vascular access and cannula position.** Panel **(A)** shows the circulatory kinetics of VV ECMO with drainage from the right internal jugular vein (RIJV) and infusion to the femoral vein (FV). The oxygenated blood from the infusion cannula (red arrow) is mixed with the venous blood in the inferior vena cava (IVC) and right atrium (RA). The mixed blood (purple arrow) flows through the lungs to the arterial side. Panel **(B)** shows the circulatory kinetics of VA ECMO with drainage from the RIJV and infusion to the femoral artery. The venous blood (blue arrow) flows through the lungs to the upper body without oxygenating the blood if the lung function is poor. Panel **(C)** shows the correct position of the draining cannula tip for VV/VA ECMO with drainage from the RIJV and infusion to the femoral vein/artery as panels **(A, B)**. The tip should be located in the upper or middle RA to drain blood with a lower O_2_ saturation from the superior vena cava (SVC). Panel **(D)** shows the tip locating the lower position than panel **(C)**, where the blood from the IVC is mostly drained. Because the blood from the IVC contains more oxygen than that from the SVC, the O_2_ saturation of the drained blood becomes higher; consequently, the efficiency of oxygenation by ECMO is decreasing. A-Ao denotes ascending aorta, D-Ao descending aorta, RV right ventricle, and FA femoral artery.

ECMO is a form of mechanical assist therapy that employs an extracorporeal blood circuit including an oxygenator and a pump. To perform standard respiratory ECMO, two vascular accesses are established, one for removal of venous blood and the other for infusion of oxygenated blood. Blood is drained from a major vein and pumped through a circuit that includes an oxygenator, which oxygenates the blood and removes carbon dioxide (CO_2_), after which the oxygenated blood is returned via the other cannula. When blood is returned to the venous side of the circulation, the procedure is known as veno-venous ECMO (VV ECMO), which provides gas exchange but cannot give cardiac support (Figure 1A). When blood is returned to the arterial side of the circulation, this is called veno-arterial ECMO (VA ECMO), and it can be employed for both gas exchange and cardiac support (Figure 1B). If the patient’s circulation is stable without high-dose inotrope therapy and echocardiography does not show right ventricular or left ventricular failure, VV ECMO should be selected. VA ECMO is associated with the potential risk of major limb vessel occlusion by the arterial cannula, as well as arterial embolism and refractory cannula site bleeding. The common reasons for selecting VA ECMO in ARDS patients are pulmonary hypertension, cardiac dysfunction associated with sepsis, and arrhythmia.

Because the main purpose of respiratory ECMO is to maintain oxygenation of the organs, adequate knowledge of oxygenation is essential for managing ECMO patients. It should be noted that “hypoxia” is different from “hypoxaemia” and that hypoxia should be avoided while hypoxaemia can be accepted [5]. Hypoxia occurs when oxygen (O_2_) delivery is insufficient to satisfy the demand of the organs [6,7]. The arterial O_2_ content (CaO_2_) is almost directly proportional to arterial O_2_ saturation (SaO_2_) × haemoglobin (Hb). For example, the CaO_2_ of a patient with SaO_2_ of 70% and Hb of 12 g/dl is higher than that of a patient with SaO_2_ of 90% and Hb of 9 g/dl. The latter situation is typical of a patient with anaemia and is unlikely to result in hypoxia [8]. Actually, hypoxia may not occur in either situation as long as cardiac output is preserved.

**Figure 2:**
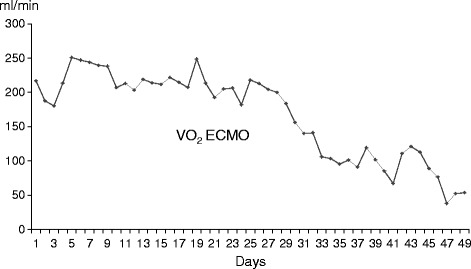
**Changes of O**_**2**_ **supplied by ECMO.** Oxygen supplied by ECMO (VO_2_ ECMO) is shown in an adult ARDS patient with H1N1 influenza. The amount of oxygen supplied decreases after the 30th day, indicating recovery of lung function. (Reproduced from Ref. [9]). VO_2_ ECMO is calculated as follows: ECC [l/min] × 1.39 [mlO_2_/gHb] × Hb [g/dl] × 10 × (outSaO_2_ − inSvO_2_), where ECC is extracorporeal circuit flow, outSaO_2_ is the saturation of arterialized blood in the returning circuit, inSvO_2_ is the venous blood saturation in the draining circuit, and Hb is the haemoglobin. The coefficient 1.39 (mlO_2_/gHb) denotes the O_2_ content (ml) per 1 g of haemoglobin.

In addition, we often want to know the amount of O_2_ supplied by ECMO. If lung function is very poor, then O_2_ consumption corresponds to the amount of O_2_ supplied by ECMO, which is determined as the difference between returning blood O_2_ content and draining blood O_2_ content multiplied by the ECMO flow rate. Thus, oxygen supply is calculated by the following formulae: ECC [l/min] × 1.39 [mlO_2_/gHb] × Hb [g/dl] × 10 × (outSaO_2_ − inSvO_2_), where ECC is the extracorporeal circuit flow rate, outSaO_2_ is the saturation of arterialized blood in the returning circuit (always 1), and inSvO_2_ is the saturation of venous blood in the draining circuit [9]. As pulmonary oxygenation improves, the amount of O_2_ supplied by ECMO decreases, which means that monitoring O_2_ supplied via ECMO can be used to assess the process of pulmonary recovery (Figure 2) [9,10]. The formulae also indicate that the efficiency of oxygenation depends on the saturation of venous blood in the draining cannula.

#### Pathophysiology of ARDS

ARDS is characterized by the acute development of bilateral lung infiltration on chest X-ray films or computed tomography scans and hypoxaemia due to any cause other than heart failure. Since the concept of ARDS was proposed in 1967, the definition has long been a topic of discussion [11]. The Berlin definition was proposed by the European Society of Intensive Care Medicine in 2011 and represents the latest consensus [1]. According to this definition, severe ARDS, which is characterized by a partial pressure of arterial O_2_/fraction of inspired O_2_ (PaO_2_/F_I_O_2_) <100 mmHg despite positive end-expiratory pressure (PEEP) >5 cmH_2_O, has a very high-mortality rate (45%).

The early phase of ARDS is characterized by inflammatory changes of the alveolar epithelium and exudation of plasma proteins into the alveoli along with neutrophils, macrophages, and erythrocytes. Fibrin and plasma proteins form a hyaline membrane on the alveolar walls that may affect lung compliance and gas exchange in addition to pulmonary inflammation. The proliferative phase of ARDS usually develops at 5 to 7 days after its onset and is characterized by proliferation of type 2 alveolar cells together with interstitial inflammation [12]. In some patients, interstitial fibrosis progresses as a result of prolonged interstitial inflammation.

While the underlying disease triggers pulmonary inflammation, the use of mechanical ventilation to treat ARDS may aggravate it. The concept of ventilator-induced lung injury has been proposed, but its mechanism is still under discussion, with a high-alveolar pressure or excessive alveolar expansion being suggested to promote such injury [13,14].

### History of adult respiratory ECMO

In 1972, Hill reported the first successful use of ECMO in an adult respiratory failure patient [2]. A 24-year-old man underwent emergency surgery for multiple fractures and aortic rupture due to a traffic accident and developed ARDS 4 days later. He recovered after being placed on VA ECMO for 75 h. This report attracted considerable attention to respiratory ECMO, and the first randomized controlled trial (RCT) was conducted in the United States between 1974 and 1977 to investigate ECMO for ARDS [15]. Patients with severe respiratory failure (either a PaO_2_ <50 mmHg for 2 h with F_I_O_2_ of 100% and PEEP >5 cmH_2_O or a PaO_2_ <50 mmHg for 12 h with F_I_O_2_ >60% and PEEP >5 cmH_2_O) were randomized to an ECMO group or a conventional treatment group. This study found no difference in 30-day survival (the primary endpoint) between the two groups, since it was 9.5% with ECMO versus 8.3% with conventional treatment. However, it should be noted that only VA ECMO was employed and high pressure, high-F_I_O_2_ ventilation was performed during ECMO.

In 1986, Gattinoni reported a single-centre observational study of low-frequency positive pressure ventilation with extracorporeal CO_2_ removal (ECCO_2_R) that employed the same entry criteria as the above-mentioned RCT and achieved a 30-day survival rate of 48.8% [16]. In this study, the ventilation rate was reduced to a minimum level, with the aim of avoiding lung damage due to repeated expansion and contraction of affected alveoli. To confirm these findings, Morris conducted a single-centre RCT, between 1987 and 1991, which enrolled 40 patients who met the same criteria as in the previous two trials [17]. The patients were randomized to an ECCO_2_R group (*n* = 21) or a conventional ventilation group (*n* = 19), and the ECCO_2_R group was treated according to the strategy reported by Gattinoni. There was no significant difference in 30-day survival, which was 42% in the ECCO_2_R group vs. 33% in the conventional ventilation group (*P* = 0.8). However, high-pressure ventilation was required in the ECCO_2_R group to maintain tidal volume and oxygenation, and ten patients (48%) from this group developed severe bleeding that led to discontinuation of ECMO in seven patients (33%). Both of these factors could have had an adverse impact on the outcome in the ECCO_2_R group.

Due to the negative findings of these RCTs, interest in adult respiratory ECMO declined around the world. However, a few departments continued to use adult respiratory ECMO, and the results gradually improved [18-20]. Peek conducted the CESAR trial of respiratory ECMO from 2001 to 2004 [21]. This RCT enrolled adult patients with severe potentially reversible respiratory failure and a Murray score >3 or a pH <7.2. Patients were excluded if they had been on aggressive mechanical ventilation for >7 days before ECMO, if they had bleeding complications incompatible with heparinization, or if they had any other condition incompatible with active treatment. Among 180 eligible patients, 90 each were randomized to an ECMO group and a conventional ventilation group. In the ECMO group, 68 patients (75%) actually received ECMO. The primary endpoint was 6-month survival without severe disabilities, which was achieved in 63% of the ECMO group compared with 47% of the conventional ventilation group (relative risk, 0.69; 95% confidence interval, 0.05 to 0.97; *P* = 0.03). The ECMO circuit used in this study could provide full oxygenation and thus allowed lung rest, which was defined as a peak airway pressure (PIP) of 20–25 cmH_2_O, PEEP of 10–15 cmH_2_O, ventilation rate of 10/min, and F_I_O_2_ of 0.3.

The H1N1 influenza pandemic occurred in 2009, the same year as publication of the CESAR trial, and The Australia and New Zealand Extracorporeal Membrane Oxygenation (ANZ ECMO) Influenza Investigators obtained favourable results with ECMO for influenza-associated ARDS [22]. They reported a survival rate of 71% for patients with a mean age of 34.4 years and Murray score of 3.8 who were on ventilation for 2 days before ECMO with a PaO_2_/F_I_O_2_ ratio of 56 mmHg, PEEP of 18 cmH_2_O, and PIP of 36 cmH_2_O. Use of ECMO during the influenza pandemic achieved varying results (survival rate: 35%–92%), probably due to differences in experience with the procedure [9,22-29]. According to the Extracorporeal Life Support Organization (ELSO) registry, the average recent survival rate is around 60%–70% for patients undergoing adult respiratory ECMO [3,4].

### Clinical use

#### Indications

While ECMO can fully replace the function of a patient’s lungs, there are various possible complications, so its use needs to be decided by assessing the balance between benefit and risk [30]. The recent literature suggests that a PaO_2_/F_I_O_2_ ratio of 70–80 mmHg, Murray score >3, and pH <7.2 provide a reasonable threshold for considering ECMO in adults with ARDS (Table 1) [4,21,25]. The absolute contraindications to ECMO are irreversible lung disease with no indication for lung transplantation and severe brain damage associated with major cerebral infarction or severe intracranial bleeding. We should decide whether or not to initiate ECMO based on the underlying disease rather than the comorbidities or the severity of multi-organ dysfunction. If the diagnosis of such an underlying disease has not been established, it is reasonable to consider ECMO.However, there is an alternative opinion of not initiating ECMO if the outcome is considered likely to be poor because it is a very expensive and labour-intensive procedure. Various criteria have been proposed as “relative contraindications” to ECMO in the literature and the ELSO guideline, such as immunosuppression, bleeding, and mechanical ventilation at high settings (F_I_O_2_ >0.9, PIP >30 mmHg) for >7 days [4,18,21,30]. It is difficult to standardize such criteria because the outcomes and available resources vary among departments or countries. While a department that has sufficient staff, financial resources, and experience might initiate ECMO in patients with a difficult background, a department possessing less resources or experience should probably not attempt it.

**Table 1:** **Indication and contraindications of ECMO for ARDS**

**Indication**	**Contraindications**
Acute reversible lung disease when conventional therapy cannot sustain life	The following underlying diseases
Severe hypoxaemia (PaO_2_/FiO_2_ ratio <80 mmHg)	Advanced cancer
Uncompensated hypercapnia (pH <7.20)	Chronic lung disease^a^
	Pulmonary fibrosis^a^
	ARDS associated with bone marrow transplantation^ab^

ECMO, extracorporeal membrane oxygenation; ARDS, acute respiratory distress syndrome; PaO_2_, arterial partial pressure of oxygen; FiO_2_, fraction of inspired oxygen

^a^If the patient is a candidate for lung transplantation, ECMO can be considered.

^b^While leukaemia is a good indication, ARDS associated with bone marrow transplantation is different. All of our patients with ARDS after bone marrow transplantation developed pulmonary fibrosis during ECMO, so we do consider that ECMO is not suitable for severe ARDS associated with bone marrow transplantation. The same result has been reported based on the data from the ELSO registry [52].

#### Cannulation

When performing cannulation, we should consider the diameter of the cannula and the position of its tip. The cannula diameter, particularly that of the draining cannula, restricts the flow rate, so it should be selected to allow adequate flow. A 23–27 Fr cannula is generally used for drainage, and a 17–21 Fr cannula is used for infusion. Low-circuit flow can occur due to incorrect positioning of the cannula tip. Another problem arises if the blood in the draining cannula has a high-O_2_ saturation, reducing the efficiency of oxygenation by ECMO (see the “Principles of respiratory ECMO” section). Cannula position should be checked regularly by chest X-ray or echocardiography because neck positioning and lung recruitment can easily shift it.

Cannulation for VV ECMO is usually performed with two single-lumen cannulas or one double-lumen cannula. If two single-lumen cannulas are employed, two vascular access points are required, which are usually the right internal jugular vein (RIJV) and a femoral vein (FV). When the draining cannula is inserted into the RIJV and the infusion cannula is placed in a FV, the draining cannula tip should be positioned in the upper or middle right atrium (RA) (Figure 1A,C). If the tip is positioned in the lower RA or the inferior vena cava (IVC), it may drain a large amount of infused oxygenated blood and cause inefficient oxygenation, which is referred to as “significant re-circulation” (Figure 1D). On the contrary, when the draining cannula is inserted into a FV and the infusion cannula is placed in the RIJV, the draining cannula tip should be positioned in the IVC. The IVC can collapse because of hypovolemia or high-abdominal pressure, and this may lead to drainage problems. The Avalon® double-lumen cannula, which became available recently, is always inserted into the RIJV [31]. Its tip should be placed in the IVC at 6–8 cm below the base of the RA so that the infusion hole (9.4 cm from the tip) is positioned in front of the tricuspid valve. Otherwise, blood may be infused into the hepatic vein or superior vena cava (SVC), causing congestive liver damage or significant re-circulation, respectively. Complications such as right ventricular perforation have also been reported [32,33].

VA ECMO should be considered for a patient with haemodynamic problems. Cannulation is normally achieved by drainage from the RA via the RIJV or a FV and infusion into a femoral artery (Figure 1B). In most cases, the heart continues to pump blood during VA ECMO, which means that less oxygenated blood from the left ventricle may circulate through the upper body while fully oxygenated blood from the circuit perfuses the lower body, so that venous O_2_ saturation may be lower in the SVC than in the IVC. In this situation, the tip of the draining cannula should be positioned in the upper or middle RA to drain the less oxygenated venous blood from the SVC (Figure 1C). If the tip is placed in the lower RA or IVC, the less oxygenated blood from the SVC may flow through the lungs to the aorta, which means that the O_2_ saturation of blood in the coronary or carotid arteries can become significantly low if the patient’s lung function is poor enough (Figure 1D).

#### Management

The only additional treatment required during ECMO is anticoagulation. Management of ECMO patients and ordinary intensive care unit (ICU) patients is essentially based on the same strategy, including minimum sedation, mobilization, conservative fluid management, and lung protective ventilation among other points. However, patients are unstable before ECMO, which means that deep sedation, paralysis, fluid overload, and high-pressure ventilation are common. ECMO can stabilize gas exchange and alleviate haemodynamic compromise, with minimum sedation and lung protective ventilation, consequently avoiding further organ damage.

There is one physiological difference between ECMO patients and ordinary ICU patients, which is related to SaO_2_. Some ECMO patients have virtually no lung function in the early phase of ARDS, so venous blood passes through the lungs without oxygenation, making it impossible to maintain the SaO_2_ above 90% even with ECMO. Although a high SaO_2_ is desirable, applying excessive pressure to the patient’s lungs is not. This is a typical dilemma that arises during ECMO. How should such patients be managed? Low SaO_2_ may not be harmful to around 70% provided that O_2_ delivery is preserved by a normal Hb and normal cardiac output [6,7,34]. Therefore, tolerating a low SaO_2_ may be a better solution than increasing the ventilator settings or performing central cannulation. Lindén reported that patients undergoing ECMO for severe ARDS remained awake when the SaO_2_ was as low as 70% and had a survival rate of 76% without long-term sequelae affecting health-related quality of life [20,35].

The same considerations apply to blood transfusion. Because of possible complications, routine blood transfusion should be avoided even for patients with low Hb, except if they have hypoxia [8,36]. For example, if an ECMO patient with a SaO_2_ of 70% and Hb of 9 g/dl develops signs of hypoxia, transfusion may be a more reasonable solution than any other intervention. The transfusion threshold varies among patients, mostly depending on the SaO_2_ and O_2_ consumption. A low-venous-O_2_ saturation is one of the clinical features of hypoxia, but symptoms probably provide the best clue as to whether or not there is a risk of hypoxic brain damage. If a patient is awake and communicating well, hypoxic brain damage may not occur even when the SaO_2_ is around 70% [20].

Ventilation at pressures high enough to damage the lungs should be avoided during ECMO. According to the ELSO guideline and CESAR trial, ventilation with a PIP of less than 25 cmH_2_O, PEEP of 5–15 cmH_2_O, and F_I_O_2_ of 0.3 is recommended during ECMO [4,21]. On the other hand, recent ARDS literature has suggested that lung protection is achieved by ventilation with tidal volume limited to as little as 6 ml per kilogram of ideal body weight and PEEP just high enough to keep the lungs open. However, the effectiveness of this approach has still not been proven, particularly during ECMO [37,38].

When the clinical state is stabilized after initiation of ECMO, waking the patient should be attempted. In ICU patients, the depth of sedation is associated with the duration of mechanical ventilation and in-hospital mortality [39], while minimizing sedation may be related to a satisfactory outcome of ECMO [20]. It is common for attempted waking to fail during the first few days because of delirium and agitation. However, the patient usually starts to adapt to the ventilator and ECMO in the following few days. Waking an ECMO patient is worth attempting because of benefits such as more stable circulation, stimulation of spontaneous breathing, a larger tidal volume, and, above all, communication with the staff and family.

Systemic anticoagulation is necessary during ECMO, which is usually achieved by infusion of unfractionated heparin and monitoring of the whole blood activated clotting time and activated partial thromboplastin time. The ELSO guideline suggests that the target activated clotting time is between 160 and 200 s and the target activated partial thromboplastin time is 1.5 times normal [4]. These values may be adjusted if the patient shows a bleeding tendency or if there is clot formation in the circuit.

#### Weaning and ceasing treatment

Weaning can be attempted after the patient has improved sufficiently with reasonable ventilator settings such as F_I_O_2_ <0.4, PIP <25 cmH_2_O, stable breathing pattern, and respiration rate <30/min [4]. With VV ECMO, weaning is achieved by simply turning off the oxygen. With VA ECMO, the flow rate is usually reduced to 1 l/min. Echocardiography is useful for accessing cardiac function or the presence of pulmonary hypertension. If circulation and gas exchange are stable with reasonable ventilator settings and low-dose inotropes, we clamp the circuit for a few minutes. If the patient develops agitation, tachypnea, and hypoxaemia, the attempt at weaning should be suspended. After weaning, patients tend to need more fluid infusion, more sedation, higher ventilator settings, and higher doses of inotropes. If patient deteriorates markedly after weaning, re-cannulation to start ECMO again should be considered.

ECMO only buys time for making a diagnosis or to allow recovery from a life-threatening underlying disease. If the patient has irreversible lung damage or severe brain damage with no chance of recovery, cessation of ECMO should be approved. However, judging an ECMO patient who is not indicated for lung transplantation to be “irreversible” is equivalent to a death sentence, so irrefutable evidence is needed. It may be impossible to make such a judgement within a few weeks after the onset of ARDS without a diagnosis. Even detection of fibrosis by computed tomography or finding pulmonary hypertension is not convincing evidence of irreversibility. When the patient does not improve by at least several weeks or 1 month after the onset of ARDS, continuing ECMO may be considered futile. Lung biopsy can be performed to confirm a diagnosis of pulmonary fibrosis. The period for which ECMO can be continued is unknown, and there have been some reports of a successful outcome after more than 1 month of treatment [10,40].

### Complications and training

In ECMO patients, severe bleeding sometimes occurs after small procedures that are safe for ordinary ICU patients. Therefore, we should consider whether even small procedures are required and should prevent bleeding complications by avoiding unnecessary procedures. For example, thoracic cavity puncture is usually safe but may occasionally lead to massive bleeding due to heparinization for ECMO.

The risk of bacteraemia and fungaemia is also high, because the blood is always in contact with artificial surfaces where bacteria and fungi can propagate easily. Cannulas can allow skin bacteria to enter the blood. There are no guidelines about prophylactic antibiotics or anti-fungal treatment for ECMO, but we should pay closer attention to this issue in ECMO patients than in ordinary ICU patients [41]. Coagulase-negative *Staphylococci* and *Candida* species are common causes of ECMO-related blood stream infection [42], and the risk of infection with *Stenotrophomonas maltophilia* and *Aspergillus* species may be increased in patient on long-term ECMO [43,44].

Circuit problems during ECMO can be fatal. Therefore, well-trained staffs are required with enough experience to ensure the safety of ECMO management. Based on data from the ELSO registry, Brodie reported that the incidence of oxygenator failure is 17.5%, while that of oxygenator clotting is 12.2%, other circuit clotting is 17.8%, cannula-related problems is 8.4%, other mechanical complications is 7.9%, and haemolysis is 6.9% [30].

Adequate staff training is essential for improving the outcome of ECMO. Water-drill training is simple and can be performed regularly. Simulation training is more complicated and expensive, but its effectiveness was reported recently [45,46]. When the Italian ECMO network was set up rapidly in 2009 because of the H1N1 influenza pandemic, ECMO simulation training was found to be effective [46]. Animals should not be used for routine training.

### Centralized ECMO and transport

ECMO is a high-risk and complicated therapy required by a small number of patients. Based on data accumulated in Paris, Combes reported that ARDS severe enough to warrant consideration of ECMO may not occur in more than five to ten cases per million population annually [47]. If all regional hospitals have an ECMO programme, each centre might only treat a few patients per year, which is not enough for the staff to maintain competence. Although the acceptable minimum number of patients is unclear, recent reports on neonatal and paediatric ECMO have suggested at least 20 cases per year are required [48,49]. To achieve this caseload, it is necessary to develop a patient transport system and perform ECMO at only selected centres. The Italian method of centralizing the management of severe ARDS patients was effective during the H1N1 influenza pandemic in 2009, being based on specific criteria and a practical algorithm from consultation to transport [25].

Because patients who need ECMO are always severely ill, conventional transport is hazardous. In the CESAR trial, 81 patients from the ECMO group were transported on mechanical ventilation and 2 patients died during transport (2.4%) [21], while a report about ECMO transport indicated that 1 out of 221 patients (0.5%) died during transport [50]. More than 670 patients have undergone ECMO transport by the ECMO Centre Karolinska transport team since 1996 and only 1 patient has died (personal data). It is impossible to statistically compare these results, but ECMO transport may be safer for ECMO candidates than transport on mechanical ventilation [50,51]. Of course, ECMO transport has to be provided by a well-trained ECMO team, and it should be available 24 h a day, 7 days a week.

## Conclusions

ECMO should be considered for patients with ARDS when they cannot survive with conventional therapy. It can stabilize gas exchange and haemodynamic compromise, thus preventing further organ damage. ECMO is not a treatment for ARDS, and the aetiology of ARDS varies. Therefore, the underlying disease should be investigated in each patient and appropriate treatment should be commenced while the patient is on ECMO. Because ECMO is complicated, training in the necessary techniques and forming a network of hospitals to manage these patients are essential. ECMO transport may be safer than transport on ventilation for transferring patients with severe ARDS to an ECMO management centre.

## Abbreviations

ARDS: Acute respiratory distress syndrome
CaO_2_: Arterial O_2_ content
CO_2_: Carbon dioxide
ECC: Extracorporeal circuit flow rate
ECCO_2_R: Extracorporeal CO_2_ removal
ECMO: Extracorporeal membrane oxygenation
ELSO: Extracorporeal Life Support Organization
Hb: Haemoglobin
ICU: Intensive care unit
IVC: Inferior vena cava
SVC: Superior vena cava
FV: Femoral vein
inSvO_2_: Saturation of venous blood in the draining circuit
outSaO_2_: Saturation of arterialized blood in the returning circuit
PaO_2_/F_I_O_2_: Partial pressure of arterial O_2_/fraction of inspired O_2_
PEEP: Positive end-expiratory pressure
PIP: Peak airway pressure
RA: Right atrium
RCT: Randomized controlled trial
RIJV: Right internal jugular vein
SaO_2_: arterial O_2_ saturation
VA ECMO: Veno-arterial ECMO
VV ECMO: Veno-venous ECMO.
